# Machine Learning Approaches in Study of Multiple Sclerosis Disease Through Magnetic Resonance Images

**DOI:** 10.3389/fimmu.2021.700582

**Published:** 2021-08-11

**Authors:** Faezeh Moazami, Alain Lefevre-Utile, Costas Papaloukas, Vassili Soumelis

**Affiliations:** ^1^Université de Paris, Institut de Recherche Saint-Louis, Institut National de la Santé et de la Recherche Médicale (INSERM) U976, Hôpital Saint-Louis, Paris, France; ^2^Université Paris-Saclay, Saint Aubin, France; ^3^Assistance Publique Hopitaux de Paris (APHP), General Pediatric and Pediatric Emergency Department, Jean Verdier Hospital, Bondy, France; ^4^Department of Biological Applications and Technology, University of Ioannina, Ioannina, Greece; ^5^Assistance Publique Hopitaux de Paris (APHP), Hôpital Saint-Louis, Immunology-Histocompatibility Department, Paris, France

**Keywords:** artificial intelligence, machine learning, multiple sclerosis, disability prediction, magnetic resonance imaging (MRI)

## Abstract

Multiple**** sclerosis (MS) is one of the most common autoimmune diseases which is commonly diagnosed and monitored using magnetic resonance imaging (MRI) with a combination of clinical manifestations. The purpose of this review is to highlight the main applications of Machine Learning (ML) models and their performance in the MS field using MRI. We reviewed the articles of the last decade and grouped them based on the applications of ML in MS using MRI data into four categories: 1) Automated diagnosis of MS, 2) Prediction of MS disease progression, 3) Differentiation of MS stages, 4) Differentiation of MS from similar disorders.

## Introduction

Multiple sclerosis (MS) is one of the main causes of acquired neurologic disability in young adults. Its prevalence varies from 5 to 300 per 100 000 (1) representing 2 to 3 million people globally (2). Disease evolution is marked by unpredictable flares of autoimmune and inflammatory central nervous system demyelination and axonal transection (3). According to the disease course, different MS stages exist. Relapsing Remitting MS (RRMS) is the most common form of MS. More than 80% will experience RRMS with neurological exacerbations separated by complete or incomplete remission (4). Secondary progressive MS (SPMS) develops from RRMS, followed by gradual neurologic deterioration not associated with acute attacks (5). Few patients will evolve into a Primary-Progressive MS (PPMS) or Progressive-Relapsing MS (PRMS) with gradual deterioration without recovery (6). Clinical presentation and detection of damage to the nervous system could help to study multiple sclerosis (MS). As MS early stage can be underdiagnosed due to non-specific clinical presentation, MRI (Magnetic Resonance Imaging) is crucial to diagnose, estimate the disease stage and predict the outcome (7). Brain lesions in MR images are an efficient imaging biomarker for multiple sclerosis diagnosis. Since detection of these lesions is laborious and time consuming and depends on radiologist experience, image processing methods based on object classification using ML learning techniques are used to apply automatic segmentation on MR images.

## Method

### Search Strategy

We conducted an electronic search on PubMed in March 2021 for the studies published from January 1st, 2011 to March 31st, 2021. The articles were searched with several appropriate keywords in combination with the Boolean operators:

Multiple sclerosis AND [ML Learning OR Artificial intelligence OR Deep learning OR Neural network] AND Magnetic resonance.

### Inclusion Criteria

We selected original publications written in English.

### Exclusion Criteria

Duplicate studies, reviews, comparative publications and case reports were excluded. Any studies not using methods of ML learning were also excluded.

In total, 106 studies were obtained that all used ML Learning models to investigate MS disease through MR images. From those, 52 were omitted according to the exclusion criteria.

## Results

After the review process of the 54 publications, we sectioned the studies into the following four categories: 1) Automated diagnosis of MS, 2) Prediction of disease progression, 3) Differentiation of MS stages, 4) Differentiation of MS disease from similar disorders.

**Figure 1:**
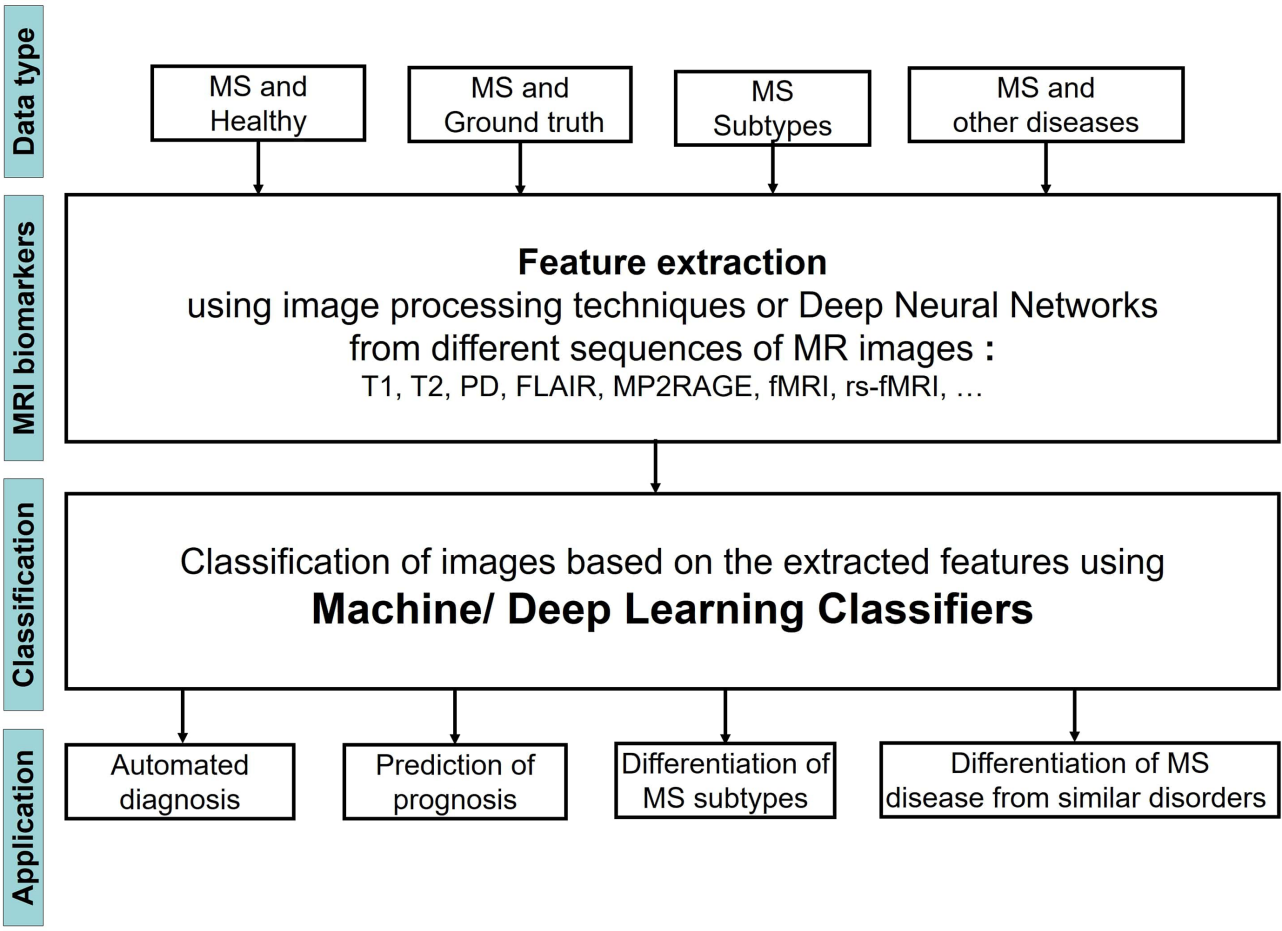
Flowchart showing the process of using ML Learning to study MS through MRI images.

**Figure 1** shows the steps of applying ML Learning classifiers on MRI images to study MS disease for the four different tasks.

We observed that the input MRI images contained different types of data including images of 1) MS patients and a healthy control group, 2) MS patients and a set of images that were segmented by an expert and considered as ground truth, 3) Patients with different stages of MS, 4) patients with MS and other diseases, very similar to MS. The images then were analyzed to extract the most important biomarkers such as whole brain atrophy biomarkers, gray matter atrophy biomarkers, spinal cord atrophy biomarkers, etc. The detection process of these lesions was carried out by using different image processing methods that are based on thresholds or various models of Deep Learning (DL). Afterwards a classifier was applied on the extracted features of the previous step to classify the MRI images into several groups and reach the aim of the study that could be one or two of the four categories: 1) Automated diagnosis of MS, 2) Prediction of MS disease progression, 3) Differentiation of MS stages, 4) Differentiation of MS from similar disorders. The classifier could be a typical ML learning model (i.e. Support Vector Machine, Random Forest, etc.) or the layer in a DL model.

The performance of both segmentation and classification steps were also quantified with various evaluation metrics. In this review, we use Accuracy (ACC), Sensitivity, Dice Similarity Coefficient (DSC), and Area Under the Curve (AUC), Root Mean Square Error (RMSE), to present performance of the models. These metrics are derived from the confusion matrix which is a two by two table formed by calculating the True Positive classified objects (TP), True Negative classified objects (TN), False Positive classified objects (FP), and False Negative classified objects (FN).

(1) $$ACC=\frac{TP+TN}{TP+TN+FN+FP}$$

(2) $$\text{Sensitivity}=\frac{TP}{TP+FN}$$

(3) $$\text{DSC}=\frac{2TP}{2TP+FN+FP}$$

(4) $$\text{RMSE}=\sqrt{\frac{1}{N}\Sigma_{i=1}^{N}{\left(\hat{y}i-yi\right)}^{2}}$$

Where N = total number of values, ŷ=predicted values, y= actual values.

AUC also measures the entire two-dimensional area underneath the entire ROC curve which plots two parameters of True Positive Rate and False Positive Rate (FPR).

In **Table 1** the summary information of the studies is described, including the quantification of the publications, the employed ML learning models and their best performances, the MRI sequences used as input images for feature extraction and the number of patients in the studies.

**Table 1:** 54 publications were grouped into 4 categories based on the application of ML Learning in MS disease.

ML application	ML model	Number of studies (*references*)	Median performance metric(min, max)	MRI Sequences (number of studies that used them)	Median sample size (min, max)
	SVM	6 (8–13)	Sensitivity: 87.5 (75, 92)% ACC: 89 (66.7, 95) %	T1 (2), T2 (2), fMRI (2), FLAIR (2), DTI (1)	19 (3, 157)
	Random Forest	1 (14)	ACC: 99.4%	FLAIR (1)	37
	Logistic Regression	1 (15)	DSC: 0.77	T2	60
	k-NN	1 (16)	Sensitivity: 77%	FLAIR	39
	Adaptive dictionary learning	1 (17)	Sensitivity: 95.8%	T1, MPRAGE, T2, PD, FLAIR	13
	CNN w/o TL method	7 (18–24)	ACC: 93 (87.12, 98.8) % DSC: 0.81 Sensitivity: 70.6%	FLAIR (4), T1 (3), T2 (2), fMRI (2), PD (1)	102 (53, 1006)
Automated diagnosis of MS	DL with TL method	4 CNN (8, 25), Resnet (26), 3D Autoencoder (27)	ACC: (83.25, 87.04) % DSC: (0.63, 0.7)	FLAIR (4), T2 (3), T1 (2)	*48 (19, 147)*
	U-net	6 (28–33)	DSC: 0.81 (0.6, 0.9)	T2 (5), FLAIR (4), T1 (2), PD (2), MP2RAGE (1)	197 (19, 1008)
	Other NNs	3 MLP, RBF, ensemble neural networks (34) MTNN (35) Cellular NN (36)	ACC: 90% Sensitivity: 77.6% DSC: 0.64	FLAIR (2), T1 (1)	45 (11, 70)
	Combination of DL and typical ML	2 Multimodal DL and Random Forest (37) DL and SVM (11)	ACC: 87.9% Sensitivity: 75%	T1 (2), T2 (1), FLAIR (1)	(44, 99)
Prediction of MS disease progression	SVM	3 (38–40)	ACC: 85.7 (70.4, 98) %	T2 (3), T1 (2), FMRI (1)	38 (25, 364)
	Random Forest	4 (39, 41–43)	ACC: 85.7 (68, 85.7) % AUC: 0.9	T1 (4), T2 (3), FMRI (2), FLAIR (1)	112 (25, 183)
	k-NN	1 (39)	ACC: 71.42%	T1, T2, rs-FMRI	25
	Naïve-Bayes	1 (39)	ACC: 71.42%	T1, T2, rs-FMRI	25
	Sparse Dictionary Learning	1 (44)	DSC: 0.77	T1, T2, PD, FLAIR	18
	Gaussian mixture	1 (45)	AUC: 0.93	T1, T2, FLAIR	20
	CNN w/o pre-training	3 (19, 46, 47)	Sensitivity: (74.2, 97) % RMSE: 0.24	T1 (2), T2 (1), PD (1), FLAIR (1)	89 (83, 1006)
	CNN with pre-training	1 (41)	ACC: 75%	T1	140
	U-net	1 (48)	DSC: 0.98	T2	553
	Other NNs	1 NN with 3 hidden layers (39)	ACC: 71.42%	T1, T2, rs-FMRI	25
	Combination of DL and typical ML	1 Combining CNN and random forest (49)	RMSE: 3	FLAIR	971
Differentiation of MS stages	LDA	1 (50)	DSC: 0.87	T1, FLAIR	105
	SVM	2 (50, 51)	DSC: 0.87 ACC: 85%	T1 (2), T2 (1), PD (1), FLAIR (1)	(105, 250)
	Naive Bayes	1 (52)	ACC: 76.5%	T1, T2	34
Differentiation of MS from similar disorders	w/o TL method CNN	1 (53)	ACC: 71.1%	T1, T2, FLAIR	338
	CNN With TL method	1 (54)	ACC: 0.75%	T2, FLAIR	88

Studies quantification and most commonly used ML Learning models are presented for each case. SVM, Support Vector Machine; NN, Neural Network; CNN, Convolutional Neural Network; TL, Transfer Learning; LDA, Linear Discriminant Analysis; k-NN, k-nearest-neighbor; rs-fMRI, resting state FMRI; DTI, Diffusion Tensor Imaging; FLAIR, Fluid-attenuated inversion recovery; PD, Proton Density; multilayer perceptron (MLP); Radial Basis Function (RBF); MTNN, Massive Training ANN.

Most of the articles studied here, have focused on automated diagnosis of MS whereas only two publications have used ML models to differentiate MRI images of MS stages. Three different strategies were used in the studies for applying ML learning models on the images. In the first strategy the lesions in the images were segmented using image processing methods, then the extracted texture features were fed to a typical ML learning model to classify the images. The second strategy used a deep Neural Network for both feature extraction from the MR images and classification of the images based on the obtained features. We also found two articles that used a combination of the first two strategies. Specifically, the first layers of a DL model extracted the features from the images, while a standard ML learning classifier was applied on the feature map obtained from the DL model.

Since the most widely used models are Neural Networks, we divided the different types of neural network into several groups to better apprehend their performances: Deep Neural Networks without pre-training, Deep Neural Networks with pre-training (TL method), U-net and Other NNs.

### Automated Diagnosis of MS

According to the McDonald and CSF criteria, the diagnosis process of MS is based on clinical presentation as well as brain and spinal cord MRI to study the dissemination of central nervous system (CNS) lesions in time and space (55). In recent years, a range of studies have proposed ML learning methods for automatic detection of central nervous system lesions from MRI on patients with MS. One of the best results for detecting T1-w and T2-w MS lesions we found in the articles, was obtained from a CNN with an accuracy of 98.8%. This model was proposed by Rocca et al. (18) and is based on four three-dimensional convolutional layers, followed by a fully dense layer after the feature extraction. It was trained with 178 scans from 268 patients.

The common problem of using CNNs is that tuning a huge number of parameters and initialize the weights are both very complex processes. In addition, such networks require a large amount of data to generalize and perform well. TL can solve these problems by employing pre-trained models, trained on a large dataset. Therefore some recent studies tried to improve DL model performance using this method. For instance Eitel et al. (8) investigated the performance of 3D convolutional neural networks (CNNs) and layer-wise relevance propagation (LRP) for the detection of brain lesions in MRI images of MS patients (n = 76) and healthy controls (n=71). They used a network that was already pre-trained on a MRI dataset from 921 patients with Alzheimer’s disease. Subsequently the pre-trained model was applied on the MS data to classify them into two groups, disease and healthy. The proposed models reached an accuracy level of 87.04% and an AUC of 0.96.

Another common DL architecture in MS image analysis is the U-net, which was developed by Ronneberger et al. (9). This model contains a fully convolutional neural network (FCNN) that includes contraction and expanding paths to perform segmentation. We found six studies that report high performance levels and applied this model on different sequences of MRI, as shown in the **Table 1**.

Furthermore, the most common typical ML model used in all studies is SVM. For instance, Zurita et al. (56) extracted fractional anisotropy maps, structural and functional connectivity from MR images as biomarkers to diagnose patients with relapsing-remitting multiple sclerosis and healthy volunteers. Then they applied SVM on the extracted features, which was able to distinguish the two groups of patients and the healthy controls with a high level of accuracy (89% ± 2%).

### Prediction of MS Disease Progression

One of the challenges in MS evaluation and the prediction of its stages is that the symptoms vary widely and as the disease worsens, new lesions become less frequent (57). In addition, the MS course in patients, even from the early stages, is characterized by a slow progression of disabilities independent of relapses (58). Automatic segmentation of MS lesions, including gadolinium-enhancing, new T2 or enlarging T2, are essential biomarkers for the progression of the disease as we as the treatment options and allow to explore the morphological changes in relation to clinical disease burden (48, 59).

Our review shows that three studies in this section applied ML to predict the progression of MS by measuring Expanded Disability Status Scale (EDSS) in the first years of disease evolution (38, 46, 49). Two of them used CNN for feature extraction and image classification, and the third one applied SVM for MR image classification based on the extracted features from the images. And also as shown in **Table 1**, SVM and Random Forests are the most commonly used models for studying the prognosis of MS patients, while both provide a high level of performance.

One of the articles in this direction assessed the influence of lesion volumes on the CNN detection performance. Coronado et al. (19) applied this model on five multispectral MRI images with different volumes of gadolinium-enhancing. They obtained the best performance by utilizing as input all five multispectral image sets, including FLAIR, T2, PD, and pre- and post-contrast T1, as well as when the enhancement size of the lesions was increased.

Youngjin Yoo et al. (41) improved the performance of their CNN model in predicting the lesions progression by using a TL method. At first they applied an unsupervised 3D convolutional deep belief network (DBN) as a pre-trained model, and then a CNN was used to extract latent MS lesion patterns. They also compared their model against the performance of Random Forests. Their CNN model resulted in an average accuracy of 75.0% in predicting the clinical conversion to definite MS within two years, while the Random Forests yielded an accuracy of 67.9%.

### Differentiation of MS Stages

In our review, we found two studies that differentiated the stages of MS patients using biomarkers such as lesion location and metabolic features and SVM classifier. Specifically, Ion-Mărgineanu et al. (50) combined lesion loads (total amount of lesion area) with clinical data magnetic resonance metabolic features to classify 87 MS patients into four groups based on the progression stages. They applied two different classification models on the extracted features: LDA and SVM with a Gaussian kernel (SVM-rbf). The highest classification (F1-score: 87%) was obtained for RRMS and SPMS after training the LDA and SVM-rbf models on clinical, lesion loads and metabolic features.

### Differentiation of MS Disease From Similar Disorders

Another important step in the diagnosis of MS is the differentiation of the disease from other similar disorders, since, despite their similarities, the treatments are greatly different. In our search we retrieved three publications related to this objective.

Two of them used different biomarkers and ML learning classifiers to distinguish MS from Neuromyelitis Optical Spectrum Disorders (NMOSD) (53, 54). The first article employed a CNN for lesion segmentation of MRI images on 213 patients with MS and 125 patients with NMOSD. This model yielded an accuracy of 71.1% in differentiating NMOSD from MS images. The second study utilized TL to pre-train two different architectures of CNN model on the ImageNet dataset (60); one model with 34 layers and one with 18 layers. The achieved accuracies of the two models were 0.75 and 0.725, respectively. In the last study Mato-Abad et al. (52) exploited a Naive Bayes classifier in order to distinguish two types of MS patients: Radiologically Isolated Syndrome (RIS) and CIS, and obtained an accuracy of 0.765, based on the morphometric measurements in MRI images.

## Conclusion

In this review we differentiated the applications of ML learning in the field of MS disease. The results show that ML can reliably support the efforts in the research field of MS disease. However, it is not possible to directly compare all the methods proposed in the related literature, since most of the works use different performance metrics to evaluate their results while the lesion volumes that were segmented in the MR images vary greatly among the studies. Nevertheless, in all ML applications, one of the most widely used models are the deep neural networks, a fact that indicates the significance of these models in the field of MS studies over the last decade. Feature extraction and selection from MR images by an expert does not allow for finding new and hidden information in the data. DL models could help to overcome this issue by extracting useful information directly from raw image data. On the other hand, the utilization of TL methods during the training process of DL models, could help to increase their performance. This approach has shown notable performance, especially in medical research where usually limited image datasets are available while image annotation by an expert is a rather tedious task.

## Author Contributions

FM, AL-U, and CP designed the project. FM studied the selected publications and wrote the manuscript. AL-U approved the medical concepts of the manuscript. CP and VS directed the project. All authors contributed to the article and approved the submitted version.

## Funding

FM was supported by the ANR DENDRITISEPSIS (ANR-17-CE15-003), and A.L-U by the Fondation pour la Recherche Médicale (FDM201806006187).

## Conflict of Interest

The authors declare that the research was conducted in the absence of any commercial or financial relationships that could be construed as a potential conflict of interest.

## Publisher’s Note

All claims expressed in this article are solely those of the authors and do not necessarily represent those of their affiliated organizations, or those of the publisher, the editors and the reviewers. Any product that may be evaluated in this article, or claim that may be made by its manufacturer, is not guaranteed or endorsed by the publisher.
